# Prognostic Impact of De-escalation Therapy in Older Adults With Breast Cancer: A Retrospective Cohort Study

**DOI:** 10.7759/cureus.93803

**Published:** 2025-10-04

**Authors:** Mako Ikeda, Yuki Kataoka, Ai Izumi, Sara Ui, Nina Odan, Sayaka Takebe, Mariko Tokiwa, Eiji Suzuki, Hirofumi Suwa

**Affiliations:** 1 Department of Breast Surgery, Amagasaki General Medical Center, Amagasaki, JPN; 2 Department of International and Community Oral Health, Tohoku University Graduate School of Dentistry, Sendai, JPN; 3 Department of Healthcare Epidemiology, Kyoto University Graduate School of Medicine/School of Public Health, Kyoto, JPN; 4 Department of Systematic Reviewers, Scientific Research Works Peer Support Group, Osaka, JPN; 5 Department of Internal Medicine, Kyoto Min-iren Asukai Hospital, Kyoto, JPN; 6 Department of Breast Surgery, Shinko Hospital, Kobe, JPN; 7 Department of Breast Surgery, Kobe City Medical Center General Hospital, Kobe, JPN

**Keywords:** adjuvant treatment, breast cancer, de-escalation therapy, geriatric oncology, recurrence-free survival

## Abstract

Background

With global population aging, breast cancer is increasingly diagnosed in older adults, and individuals aged 70 years and above now account for over 30% of new cases. Standard treatments, including surgery, radiation, endocrine therapy, and chemotherapy, are effective in reducing recurrence and improving survival. However, older patients remain underrepresented in clinical trials, resulting in limited evidence specific to this population. In clinical practice, de-escalated adjuvant therapies are often employed in older patients, but their prognostic impact remains uncertain. This study aimed to evaluate recurrence outcomes according to treatment intensity in elderly breast cancer patients.

Methods

We conducted a retrospective cohort study of 399 breast cancer patients aged ≥70 years who underwent surgery at Kobe City Medical Center General Hospital between July 2011 and March 2023. Patients were classified as receiving either standard or de-escalated adjuvant therapy, with standard therapy defined according to the 2022 Japanese Breast Cancer Clinical Practice Guidelines. Clinical data, including comorbidities, stage, and tumor subtype, were collected. Recurrence-free survival (RFS) was assessed using Cox proportional hazards models, adjusting for clinical covariates. Multiple imputation was performed to address missing data. Subgroup analyses were conducted by age, stage, estrogen receptor (ER) status, and human epidermal growth factor receptor 2 (HER2) status.

Results

The cohort included 399 patients (median age: 75 years) with a median follow-up of 56 months (interquartile range: 27-89). Only five recurrences (1.3%) were observed. In the unadjusted analysis, the median RFS was 46 months in the standard treatment group and 62 months in the de-escalation group. After adjustment with multiple imputation, the median RFS was not reached in either group. The hazard ratio for recurrence in the standard versus de-escalation group was 0.094 in the unadjusted analysis and 1.51 after adjustment. Notable differences were observed between groups with respect to comorbidities, disease stage, and tumor subtype.

Conclusions

De-escalated adjuvant therapy may be an appropriate option for selected elderly breast cancer patients, particularly those with favorable disease profiles. The very low recurrence rate observed in this cohort supports individualized treatment strategies that balance efficacy with tolerability. Prospective studies are warranted to confirm these findings and to inform evidence-based decision-making in geriatric oncology.

## Introduction

Breast cancer is one of the most prevalent malignancies among older adults worldwide, and its incidence continues to rise in parallel with global population aging. According to a 2021 analysis from the Global Burden of Disease Study, the age-standardized incidence rate among individuals aged ≥70 years was approximately 107 per 100,000, a slight increase from 104 per 100,000 in 1990, with notable elevations among women aged 70-74 years (from 86.3 to 90 per 100,000) [1]. This group now accounts for more than 30% of all breast cancer diagnoses globally.

Standard treatments, including surgery, radiation therapy, endocrine therapy, and chemotherapy, are central to controlling both local and systemic diseases and have been shown to reduce recurrence and improve survival in large clinical trials. However, most pivotal trials have enrolled relatively few older adults, particularly those aged ≥70 years, resulting in a knowledge base largely derived from younger, healthier populations. For example, a systematic Food and Drug Administration (FDA) review of cancer drug registration trials between 1985 and 2012 found that only 7% of participants in adjuvant breast cancer trials and 15% in metastatic trials were aged ≥70 years [2]. Similarly, Hutchins et al. reported that patients aged ≥65 years represented only 13% of participants in breast cancer adjuvant trials and 12% across all cancer trials, with those aged ≥75 years comprising just 4% [3]. More recent real-world data from the French Epidemiological Strategy and Medical Economics (ESME) cohort confirmed this underrepresentation, with only 4% of patients aged ≥70 years enrolled in first-line metastatic breast cancer trials, compared to 10.5% among younger patients [4].

In contrast, older patients often present with multiple comorbidities and functional impairments, making them more vulnerable to treatment-related toxicity and adverse impacts on quality of life. Consequently, in real-world practice, de-escalated therapy is often used in this population in place of guideline-recommended regimens. However, the prognostic implications of such approaches remain unclear. Clinical trials often fail to represent real-world older adults: for instance, Sedrak et al. found that fewer than 10% of trial participants were aged ≥75 years, despite this group comprising more than 30% of the overall cancer population [5].

The present study addresses this gap by conducting a retrospective cohort analysis of breast cancer patients aged ≥70 years, adjusting for clinical factors such as comorbidities, surgical approach, stage, and tumor subtype. By evaluating recurrence outcomes across varying levels of treatment intensity in a real-world setting, this study aims to generate evidence to guide individualized treatment strategies in elderly breast cancer patients.

## Materials and methods

Study design

This retrospective cohort study was conducted at Kobe City Medical Center General Hospital, Kobe, Japan, in accordance with the Strengthening the Reporting of Observational Studies in Epidemiology (STROBE) guidelines [6]. The study protocol was approved by the Institutional Review Board of Kobe City Medical Center General Hospital (approval number: zn231010).

Study population

Inclusion criteria were patients aged 70 years and older diagnosed with breast cancer between July 1, 2011, and March 31, 2023. Exclusion criteria included a prior history of breast cancer, receipt of chemotherapy for breast cancer or other malignancies before the study period, and cases without surgical treatment.

Data collection

Clinical data were obtained from electronic medical records, including operative reports, laboratory findings, and histopathology results, covering the study period.

Outcome

The primary outcome was recurrence-free survival (RFS), defined as the time from curative surgery to the first recurrence or death from any cause. We selected surgery as time zero to avoid immortal-time bias, because adjuvant treatment was assigned postoperatively. Recurrence was evaluated through routine imaging during scheduled outpatient follow-up visits.

Study size

A total of 566 patients aged 70 years and older were identified during the study period. The target sample size of approximately 500 was estimated based on the anticipated number of eligible cases, without formal statistical calculation. Of the initial cohort, 23 patients with stage IV disease and 167 with incomplete medical records were excluded, leaving 399 patients in the final analysis.

Definition of standard therapy and de-escalation therapy 

Based on the Japanese Breast Cancer Society guidelines, treatment categories were defined as follows:

Local Therapy

Standard treatment was defined as radiotherapy following breast-conserving surgery, while omission of radiotherapy was defined as de-escalation.

Hormone Receptor-Positive Breast Cancer

Standard treatment was defined as endocrine therapy, while no treatment was considered de-escalation.

Human Epidermal Growth Factor Receptor 2 (HER2)-Positive Breast Cancer

Standard treatment was defined as anti-HER2 therapy in combination with chemotherapy, while anti-HER2 therapy alone or no treatment was considered de-escalation.

Triple-Negative Breast Cancer

Standard treatment was defined as intravenous chemotherapy with both anthracycline- and taxane-based regimens, while treatment with only one of these agents, oral chemotherapy, or no treatment was considered de-escalation.

Receiving a reduced number of chemotherapy cycles was defined as de-escalation therapy.

Statistical analysis

Differences in recurrence and overall survival between de-escalated and standard therapy were analyzed. Standard therapy was defined according to the 2022 edition of the Japanese Breast Cancer Clinical Practice Guidelines, with all other regimens classified as de-escalated. Age, stage, cancer subtype (estrogen receptor (ER)/progesterone receptor (PgR)/HER2), and comorbidities were included as covariates.

ER, PgR, and HER2 status were assessed by immunohistochemistry (IHC) in accordance with the 2018 American Society of Clinical Oncology (ASCO)/College of American Pathologists (CAP) guidelines. ER and PgR were considered positive if ≥1% of tumor cells showed nuclear staining. HER2 was considered negative if scored as 0 or 1+ and positive if scored as 3+. For tumors with a HER2 score of 2+, fluorescence in situ hybridization (FISH) was performed, and HER2 positivity was defined as a gene copy number ≥4.

Descriptive analyses were conducted using summary statistics. Survival curves were estimated with the Kaplan-Meier method, and confidence intervals were calculated using Greenwood's formula. Differences in survival curves were compared using the log-rank test. Cox proportional hazards models were applied for covariate adjustment. Analyses were performed using R Version 4.3.3 in the Posit Cloud environment (RStudio IDE Version 2024.4.2.764.1, R Foundation for Statistical Computing, Vienna, Austria).

Subgroup analyses were performed by age, stage, ER status, and HER2 status. Interaction terms between treatment type and each subgroup variable were incorporated into the Cox models. Missing data were assumed to be missing at random (MAR) and were handled using multiple imputation by chained equations (MICE). Continuous variables were imputed by predictive mean matching, while binary, unordered categorical, and ordered categorical variables were imputed using logistic, polytomous logistic, and proportional-odds models, respectively. The imputation model included all exposures, outcomes, confounders, and auxiliary predictors of missingness. Fifty imputations were generated with 20 iterations each, and convergence was confirmed via trace and density plots. Each imputed dataset was analyzed with the prespecified regression model, and estimates were pooled using Rubin's rules.

In the complete-case analysis, standard therapy was associated with a lower risk of recurrence (HR 0.094; 95% CI 0.013-0.708; p=0.022). Complete-case analyses and missing-not-at-random (MNAR) sensitivity checks (±0.5 SD) were consistent with the main results. Reporting followed STROBE guidelines and current recommendations on multiple imputation.

Bias

To minimize bias, stringent inclusion and exclusion criteria were applied to reduce selection effects. Multivariable Cox proportional hazards models adjusted for key confounders, including age, stage, cancer subtype, and comorbidities. Recurrence was consistently assessed through imaging during outpatient follow-up, and outcome definitions were standardized across all groups.

## Results

**Figure 1:**
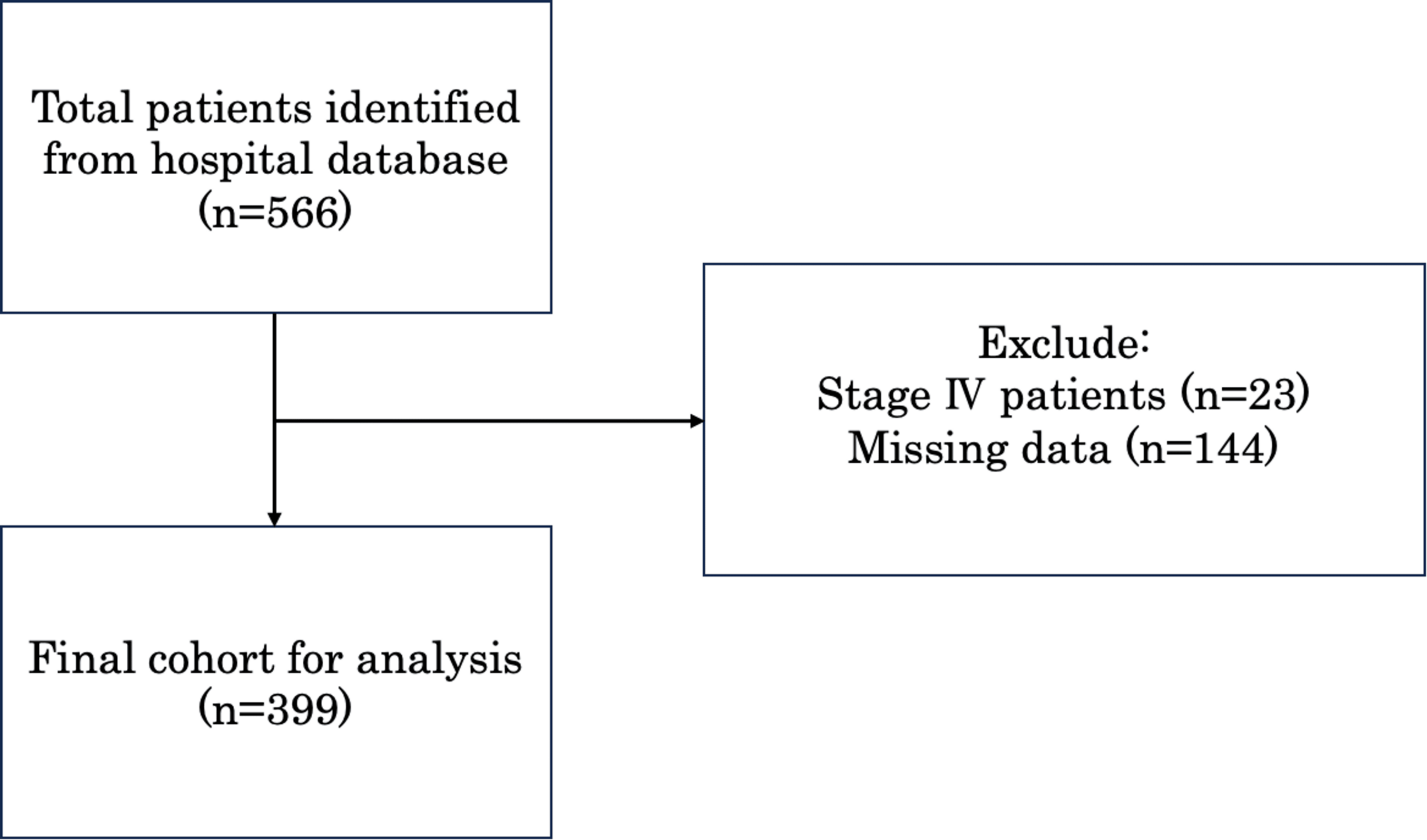
Patient selection flow diagram (July 1, 2011-March 31, 2023)

Between July 1, 2011, and March 31, 2023, 566 patients aged ≥70 years at diagnosis were identified at Kobe City Medical Center General Hospital. After excluding 23 patients with stage IV disease and 144 with incomplete medical records, 399 patients were included in the final analysis. The median follow-up was 56 months (IQR: 27-89) (Figure 1).

The median age was 75 years (IQR: 72-80), and the median body mass index (BMI) was 22.8 kg/m² (IQR: 20.5-25.5). The cohort was predominantly female (99.9%). Patients with stage 0 to stage IIIC breast cancer were included; 18.8% had stage 0 disease, and 74.6% had stage I-IIIA disease. Regarding tumor subtypes, 64.4% were ER-positive, 41.4% were PgR-positive, and 23.3% were HER2-positive. Patient characteristics are summarized in Table 1.

**Table 1:** Patient characteristics ER: estrogen receptor; PgR: progesterone receptor; HER2: human epidermal growth factor receptor 2

	Median (IQR)		N=399 (%)
Age	75 (72-80)		-
BMI (kg/m^2^)	22.8 (20.5-25.5)		-
Sex	Female		398 (99.9)
	Male		1 (0.01)
Stage	Stage 0		75 (18.8)
	Stage I		178 (44.7)
	Stage IIA		85 (21.3)
	Stage IIB		28 (7)
	Stage IIIA		7 (1.6)
	Stage IIIB		21 (5.3)
	Stage IIIC		5 (1.3)
Subtype	ER	Positive	257 (64.4)
		Negative	91 (22.8)
		Missing	51 (12.8)
	PgR	Positive	164 (41.1)
		Negative	153 (38.3)
		Missing	82 (20.6)
	HER2	Positive	93 (23.3)
		Negative	91 (22.8)
		Missing	215 (53.9)
Comorbidities	Depression		6 (1.5)
	Schizophrenia		6 (1.5)
	Myocardial infarction		7 (1.8)
	Congestive heart failure		19 (4.8)
	Peripheral arterial disease		3 (0.8)
	Dementia		6 (1.5)
	Cerebrovascular disease		17 (4.3)
	Lung disease		1 (0.3)
	Collagen disease		3 (0.8)

**Figure 2:**
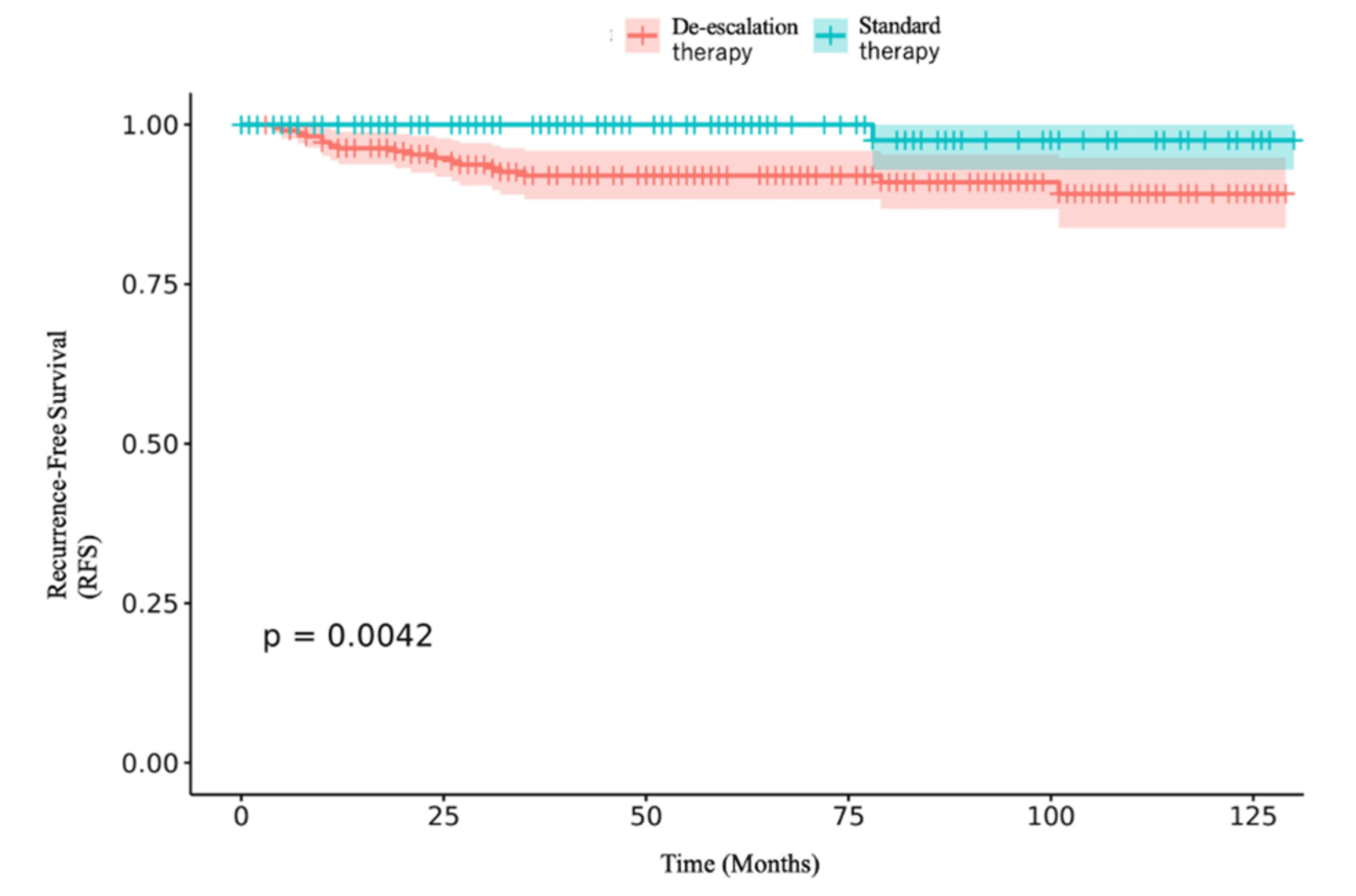
Kaplan-Meier survival curves stratified by standard treatment

Of the 399 patients, 157 received standard therapy, and five (1.3%) experienced recurrence. Kaplan-Meier survival curves, compared using the log-rank test, are shown in Figure 2.

In the unadjusted analysis, the median RFS was 46 months in the standard therapy group and 62 months in the de-escalation group. Standard therapy was associated with a crude hazard ratio of 0.094 (95% CI 0.013-0.708). After adjustment for clinical covariates, the hazard ratio was 1.51 (95% CI 0.95-2.39) (Table 2).

**Table 2:** Multivariate analysis of factors associated with outcomes

Variable	Hazard ratio	95% CI			P-value
Standard therapy vs. de-escalation therapy (unadjusted analysis)	0.094	0.013-0.708			0.022
						Standard therapy vs. de-escalation therapy	1.51	0.95-2.39			0.084

## Discussion

Summary of results

This retrospective cohort study found that disease recurrence was infrequent among elderly breast cancer patients and the association between treatment intensity and recurrence risk varied depending on adjustment for covariates. In unadjusted analyses, standard therapy appeared to be associated with a markedly lower risk of recurrence; however, after adjusting for clinical variables such as age, stage, and tumor subtype, this difference diminished, suggesting that baseline patient characteristics may partly explain the observed outcomes. These findings emphasize the importance of individualized treatment planning and highlight the challenges of interpreting observational data, particularly in older populations with heterogeneous clinical profiles.

Comparison with previous studies

The paradigm of "less is more" is gaining momentum in geriatric oncology, with growing interest in de-escalating treatment to minimize toxicity while maintaining efficacy. The RESPECT (Randomized controlled trial of trastuzumab monotherapy versus trastuzumab plus chemotherapy in older patients with HER2-positive breast cancer) study, a prospective cohort study conducted alongside a randomized trial, provided important evidence supporting this approach [7]. It showed that elderly patients with HER2-positive breast cancer treated with trastuzumab monotherapy had outcomes comparable to those receiving trastuzumab plus chemotherapy, suggesting that trastuzumab alone may be a reasonable option in this setting. Similarly, our retrospective study found no difference in recurrence outcomes between standard and de-escalated therapies after adjustment for clinical covariates.

By contrast, earlier trials such as CALGB (Cancer and Leukemia Group B) 49907 demonstrated that fit elderly patients can benefit from standard chemotherapy regimens [8]. Our findings did not confirm a clear advantage of standard therapy over de-escalated approaches. This discrepancy may be explained by differences in study design and patient selection. CALGB 49907 enrolled elderly patients with good performance status who were candidates for chemotherapy, whereas our cohort represented a broader real-world population with greater heterogeneity in health status and comorbidities. These differences likely influenced both treatment allocation and tolerance, potentially accounting for the lack of an observed benefit from standard chemotherapy in our study.

Clinical implications

For elderly patients with breast cancer, clinicians may consider de-escalating adjuvant therapy, particularly in those with favorable risk profiles, to reduce treatment-related toxicity without compromising oncologic outcomes. Older adults frequently present with comorbidities and diminished physiological reserves, heightening their vulnerability to adverse effects from standard chemotherapy. In patients with low-risk disease features, such as early-stage tumors, favorable subtypes, or strong hormonal responsiveness, a less intensive approach may strike a more appropriate balance between efficacy and tolerability [9-11]. Therapy should be individualized according to functional status, life expectancy, and patient preferences, ideally guided by comprehensive geriatric assessment and shared decision-making [12,13]. In addition, discussing each case in a multidisciplinary committee to evaluate the appropriateness of de-escalation may help optimize both quality of life and survival outcomes.

Generalizability

The generalizability of our findings may be limited by regional and ethnic factors. Given the single-center design and exclusive inclusion of Japanese patients aged 70 and older, these results may not be directly applicable to more diverse populations. They may be particularly relevant to other Asian countries with comparable life expectancy, genetic backgrounds, and healthcare infrastructures; however, caution is required when extrapolating to Western populations, where comorbidities, treatment preferences, and healthcare delivery systems differ considerably [12].

Limitations

This study has several limitations. First, its retrospective design introduces potential for residual confounding. Second, patients with incomplete medical records were excluded, particularly those missing data on tumor subtypes such as HER2 status and details on adjuvant radiotherapy. In addition, information regarding chemotherapy dose reduction was not available; therefore, dose-related details could not be fully assessed, which represents another limitation of this study. Cases with HER2 2+ status were excluded due to indeterminate HER2 classification, reducing the number of cases with subtype data and potentially introducing bias into subtype distribution. Such reductions may have influenced the observed outcomes, although it remains unclear whether they would have strengthened or weakened the association with de-escalated therapy.

In contrast, radiotherapy was less prone to subtype-related bias, as its use was limited to patients undergoing breast-conserving surgery [14]. Given that prognosis is more directly affected by tumor subtype than by radiotherapy, the missing radiotherapy data were expected to have minimal impact on overall study results. Definitions of RFS vary (e.g., diagnosis-based time origin), and in this study, we defined RFS from the date of surgery. While this approach may not capture events occurring before surgery, it helps reduce immortal-time bias when evaluating adjuvant treatment effects.

Moreover, detailed information on baseline characteristics such as comorbidities and the specific clinical rationale underlying de-escalation decisions was not uniformly available, which limited our ability to fully characterize the two groups and the context in which treatment strategies were chosen. Addressing these gaps will require prospective studies with systematic and standardized data collection.

To address these limitations, future prospective studies with standardized data collection, including complete molecular subtype classification and detailed treatment information, are needed to validate our findings and more accurately evaluate the impact of de-escalated therapy in elderly breast cancer patients. In particular, randomized prospective trials directly comparing standard therapy with de-escalated therapy would provide more definitive evidence, as retrospective designs are inherently limited in establishing causal relationships.

## Conclusions

This study suggests that de-escalated adjuvant therapy may represent a viable treatment option for elderly patients with breast cancer, particularly those with favorable disease characteristics. Although recurrence was rare in our cohort, the association between treatment intensity and outcomes may have been influenced by baseline clinical factors. These findings support ongoing efforts to individualize treatment strategies in geriatric oncology. Further prospective studies incorporating comprehensive clinical and molecular data are warranted to validate these results and better inform treatment decisions in this population.
